# Systematic Genomic Surveillance of SARS-CoV-2 Virus on Illumina Sequencing Platforms in the Slovak Republic—One Year Experience

**DOI:** 10.3390/v14112432

**Published:** 2022-11-02

**Authors:** Diana Rusňáková, Tatiana Sedláčková, Peter Radvák, Miroslav Böhmer, Pavol Mišenko, Jaroslav Budiš, Silvia Bokorová, Nikola Lipková, Michaela Forgáčová-Jakúbková, Tomáš Sládeček, Jozef Sitarčík, Werner Krampl, Michaela Gažiová, Anna Kaliňáková, Edita Staroňová, Elena Tichá, Terézia Vrábľová, Lucia Ševčíková, Barbora Kotvasová, Lucia Maďarová, Soňa Feiková, Kristína Beňová, Lenka Reizigová, Zuzana Onderková, Dorota Ondrušková, Dušan Loderer, Mária Škereňová, Zuzana Danková, Katarína Janíková, Erika Halašová, Elena Nováková, Ján Turňa, Tomáš Szemes

**Affiliations:** 1 Comenius University Science Park, 841 04 Bratislava, Slovakia; 2 Department of Molecular Biology, Faculty of Natural Sciences, Comenius University, 842 15 Bratislava, Slovakia; 3 Geneton Ltd., 841 04 Bratislava, Slovakia; 4 Slovak Centre of Scientific and Technical Information, 840 05 Bratislava, Slovakia; 5 Public Health Authority of the Slovakia, 826 45 Bratislava, Slovakia; 6 Regional Public Health Authority with the Seat in Banská Bystrica, 975 56 Banská Bystrica, Slovakia; 7 Regional Public Health Authority with the Seat in Trenčín, 911 01 Trenčín, Slovakia; 8 Regional Public Health Authority with the Seat in Košice, 040 11 Košice, Slovakia; 9 Biomedical Centre Martin, Jesseniuss Faculty of Medicine, Comenius University, 036 01 Martin, Slovakia; 10 Department of Microbiology and Immunology, Jessenius Faculty of Medicine in Martin, Comenius University, 036 01 Martin, Slovakia

**Keywords:** SARS-CoV-2, COVID-19, Slovak Republic, NGS, genomic surveillance

## Abstract

To explore a genomic pool of severe acute respiratory syndrome coronavirus 2 (SARS-CoV-2) during the pandemic, the Ministry of Health of the Slovak Republic formed a genomics surveillance workgroup, and the Public Health Authority of the Slovak Republic launched a systematic national epidemiological surveillance using whole-genome sequencing (WGS). Six out of seven genomic centers implementing Illumina sequencing technology were involved in the national SARS-CoV-2 virus sequencing program. Here we analyze a total of 33,024 SARS-CoV-2 isolates collected from the Slovak population from 1 March 2021, to 31 March 2022, that were sequenced and analyzed in a consistent manner. Overall, 28,005 out of 30,793 successfully sequenced samples met the criteria to be deposited in the global GISAID database. During this period, we identified four variants of concern (VOC)—Alpha (B.1.1.7), Beta (B.1.351), Delta (B.1.617.2) and Omicron (B.1.1.529). In detail, we observed 165 lineages in our dataset, with dominating Alpha, Delta and Omicron in three major consecutive incidence waves. This study aims to describe the results of a routine but high-level SARS-CoV-2 genomic surveillance program. Our study of SARS-CoV-2 genomes in collaboration with the Public Health Authority of the Slovak Republic also helped to inform the public about the epidemiological situation during the pandemic.

## 1. Introduction

A novel Coronavirus disease (COVID-19), which appeared in Wuhan city of China in late 2019, was declared a global pandemic by the World Health Organization (WHO) on 11th March 2020 [1]. The statistics to date (5 October 2022) record more than 615 million confirmed infections and more than 6.5 million deaths [2]. The first SARS-CoV-2 genome was determined in January 2020 [3] and next-generation sequencing (NGS) has been the most common way to identify and track emerging nucleotide changes with possible effects on infectivity or pathogenicity [4,5]. Sequencing data have shown significant mutational changes several times during the COVID-19 pandemic [6]. These data are increasingly studied to understand the potential links between transmission dynamics, pathogenicity, diagnostic performance, vaccine efficacy and immune escape [7].

The genome size of the SARS-CoV-2 virus is approximately 29.9 Kb [8]. Mutation can occur in any of the regions of the genome. However, most changes have little to no impact on the virus properties. Some changes, especially in structural proteins, affected the properties of the virus, resulting in different lineages [9]. Nomenclature systems from the Global Initiative on Sharing All Influenza Data (GISAID) [10], Nextstrain [11] and Pango [12] are currently used to name and track SARS-CoV-2 genetic lineages. WHO has introduced and has recommended a uniform nomenclature using letters of the Greek Alphabet and has divided the lineages into three categories: Variants of Concern (VOCs), Variants of Interest (VOIs) and Variants under Monitoring (VUMs). Lineages included in VOC have changed phenotypically compared to a reference isolate or have a genome with mutations that lead to amino acid changes. There is clear evidence to suggest a significant effect on transmissibility, severity and immune response likely linked with the new epidemiological situation. For example, Alpha, Delta and Omicron variants classified as VOC, whose new mutations in the genome led to the higher virus transmission, caused new waves with an increase in positive cases globally [9]. Lineages for which there is at least preliminary scientific evidence that they could affect transmission, severity or immune escape—which could also affect the epidemiological situation—are designated as VOI [13]. VUM are variants of SARS-CoV-2 whose genomic mutations could affect the characteristics of the virus and pose a risk in the future; however, the evidence of impact is unclear. Monitoring of such variants are in place until new evidence about the effects of mutations on overall virus fitness is known [9].

For monitoring of novel or emerging variants, detection at a prevalence of 0.1% to 1.0% is the recommended minimum by the European Center for Disease Prevention and Control (ECDC). Therefore, ECDC recommends as optimal sequencing rate of 5% of all COVID-19 positive samples [14]. Many countries worldwide have launched national viral genome surveillance [15,16,17,18], which allows tracking of the diversity of SARS-CoV-2 viruses circulating in the world in real-time. Viral genomic surveillance needs to be of global interest to be effective. Unfortunately, the monitoring of SARS-CoV-2 remains inconsistent [19]. The limited genome sequencing intensity may negatively affect the identification and thus late response to new viral lineages with shifted epidemiological and antigenic characteristics [20].

Here we describe the Slovak Republic (SR) response to COVID-19 by forming an expert workgroup for genomics surveillance under the Ministry of Health and coordinated by the Public Health Authority of the Slovak Republic. Launching of the Slovak national SARS-CoV-2 sequencing program has contributed to the worldwide endeavor to monitor the development of the COVID-19 pandemic. This study represents the major results obtained by six out of seven participating laboratories using Illumina sequencing technology and the unified analysis from the program launch in March 2021 until March 2022.

## 2. Material and Methods

### 2.1. SARS-CoV-2 Samples

Nasopharyngeal swabs were collected from medical subjects for RT-PCR routine testing of SARS-CoV-2, which were widespread throughout the country. This population represented the entire range of the entire clinical spectrum. Swabs were dipped into 3 mL of inactivated virus transport medium and transported under refrigeration (2–8 °C) to the central laboratory PHA SR located in Bratislava. Samples for genome sequencing were selected both randomly and selectively (based on positive travel anamnesis, positive after vaccination, atypical course of COVID-19, reinfection, close contacts or certain severe case subgroups). All samples that were chosen had an RT-PCR cycle threshold (CT) ≤ 30, which was set up as a limit for successful genome sequencing.

### 2.2. RNA Isolation

All samples selected for sequencing had nucleic acid freshly extracted from the primary sample source independently of the material extracted for the initial RT-PCR testing. RNA isolation was performed at 4 workplaces—the Public Health Authority of the Slovak Republic in Bratislava (PHA SR), the Regional Public Health Authority in Banská Bystrica (RPHA BB), the Regional Public Health Authority in Trenčín (RPHA TN) and the Regional Public Health Authority in Košice (RPHA KE). Samples for the Comenius University Science Park in Bratislava (CU SP) and the Jessenius Faculty of Medicine of Comenius University (JFM CU) in Martin sequencing centers were isolated at PHA SR in Bratislava. The workplaces extracted viral RNA from nasopharyngeal swab samples using RNAdvance Viral RNA Extraction Kit (Beckman Coulter, CA, USA), QIAamp Viral RNA Mini Kit (QIAGEN GmbH, Hilden, DE, USA) and Quick-RNA™ Viral Kit (Zymo Research Corp., Irvine, CA, USA). They followed the manufacturer’s protocol with the following small exception to the Quick-RNA™ Viral Kit: for the first step, the addition of 100 μL of DNA/RNA Shield™ was skipped. RNA isolation was performed on a plate and then stored in a 96-well plate at −80 °C. Subsequently, the plates with isolated RNA were processed by the appropriate sequencing center.

### 2.3. NGS Sequencing

Six out of seven involved genomic laboratories performed sequencing on Illumina sequencing platforms. These were CU SP, PHA SR, JFM CU, RPHA BB, RPHA TN and RPHA KE. Whole-genome sequencing libraries of the SARS-CoV-2 were prepared manually in 96-well plates (95 samples, 1 non-template control) according to the Illumina COVIDSeq Test protocol (Illumina Inc., San Diego, CA, USA). After cDNA synthesis and amplification using COVIDSeq™ V3 Primer Pool (replaced by new COVIDSeq™ V4 Primer Pool from December 2021) based on the ARTIC protocol, PCR amplicons were tagmented using IDT^®^ for Illumina PCR Unique Dual Indexes Set 1–4 (384 Indexes). Libraries were purified and pooled following the manufacturer’s guidelines (Illumina Inc., San Diego, CA, USA). Library pools were quantified on a Qubit 3.0 fluorometer (Invitrogen Inc., Waltham, MA, USA) and then normalized to 4 nM. All laboratories used Illumina platforms (Illumina Inc., San Diego, CA, USA)—three laboratories (RPHA BB, RPHA TN and RPHA KE) used MiniSeq, PHA SR—MiSeq, JFM CU—NextSeq 550 and CU SP—NextSeq 500. At the beginning of the national sequencing project, the sequencing parameters for each platform were as follow: 2 × 300 bp paired-end for MiSeq, 2 × 75 bp paired-end for MiniSeq, 2 × 36 bp or 2 × 74 bp paired-end for NextSeq 500, 2 × 74 bp paired-end for NextSeq 550. They were later unified for all sequencing platforms for 2 × 74 bp paired-end reads.

### 2.4. Bioinformatic Analysis

Detailed information about the pipeline is available in Goga et al., 2021 [21]. Briefly, the reads were pre-processed with the trimming step using the Cutadapt tool [22]. Subsequently, the reads were subjected to a decontamination process in which the fragments of human RNA were eliminated. In the mapping step, we employed BWA [23] to map the reads to the SARS-CoV-2 reference genome and SAMtools [24] to sort and index the generated SAM/BAM files. Variant calling and construction of consensus sequences were performed by the BCFtools [25]. NextClade [26] was then used to check quality of consensus sequences. Finally, sequences with sufficient coverage (at least 3 across more than 90% of the genome) were uploaded to public repositories—European Nucleotide Archive [27] and GISAID [28]. All computational analyses were written and executed using the SnakeLines framework [29,30] and are independent of the sequencing reads length.

In February 2022, an integrated sample data, metadata management and processing information system was launched to optimize all data transfers within sequencing processes, to allow unified analysis, verification and batch uploading to repositories. The previously-mentioned pipeline became the data analysis module of this system. This integrated system for national COVID-19 sequencing has an abbreviated name—NarCoS (from Národné COVID-19 Sekvenovanie—National COVID-19 sequencing).

### 2.5. SARS-CoV-2 Phylogeny

Samples metadata obtained from public database GISAID were filtered to select 165 genomes representing unique lineage detected between March 1, 2021, and March 31, 2022. Each genome represents one Pango lineage detected with the national surveillance program. All 165 full-length SARS-CoV-2 genomes were retrieved from GISAID. Consensus sequences were visualized on the backbone of the global SARS-CoV-2 lineage tree using the interactive Nextclade v1.14.1 platform [26,30,31].

## 3. Results

### 3.1. SARS-CoV-2 Genome Surveillance Program

To summarize and review Slovak republic SARS-CoV-2 pandemic data, we analyzed a set of sequenced samples generated during a specific period. Over 1.4 million (*n* = 1,407,713) positive SARS-CoV-2 cases were detected and confirmed by RT-PCR over thirteen months [32]. Out of all positive samples, COVID-19 dedicated laboratories isolated and sent randomly and/or specifically selected samples (*n* = 33,024) for sequencing and SARS-CoV-2 variant determination, which represents 2.35%. It needs to be said that the actual percentage number is slightly larger, as we here describe data generated only by collaborating laboratories using Illumina sequencing and the analysis described in the methods. Of all suitable samples, 93.2% were successfully sequenced and analyzed (*n* = 30,793) to resolve the variant of SARS-CoV-2 virus. Finally, 84.8% of the high-quality consensus sequences (*n* = 28,005) met all criteria for submission to the GISAID repository to be shared with the scientific community.

### 3.2. Sample Characteristics

**Figure 1:**
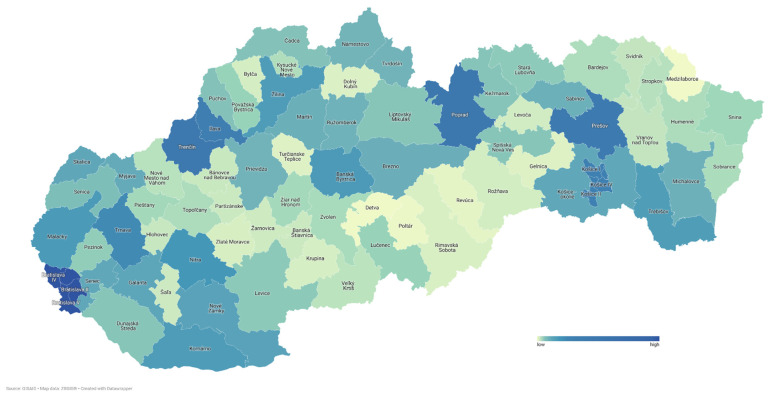
Map of quantity and origin of SARS-CoV-2 samples in SR. Heterogeneity of sequenced samples in districts range from 21 from Medzilaborce up to 3894 in Bratislava (created with datawrapper.de). List of all districts with exact numbers of sequenced samples are in the Supplementary data Table S1.

This study analyzed data from SARS-CoV-2 positive cases isolated and confirmed in the SR which were submitted to GISAID and are publicly available. Individual samples and their corresponding metadata were selected based on the date of sample collection and geographic location in the Slovak Republic (*n* = 32,486). Samples were also filtered based on originating and sequencing laboratory information to a final dataset for further analysis and statistics (*n* = 28,005). The remaining samples (*n* = 4481) were not sequenced in the six mentioned laboratories. Out of the final dataset (*n* = 28,005), 54.13% were female samples (*n* = 15,158) with an average age of 40, and 45.87% of samples (*n* = 12,847) were male with an average age of 39. Most of the samples were collected randomly through testing centers from all districts of the SR (Figure 1).

### 3.3. Sequencing Volume of the Surveillance Program

**Figure 2:**
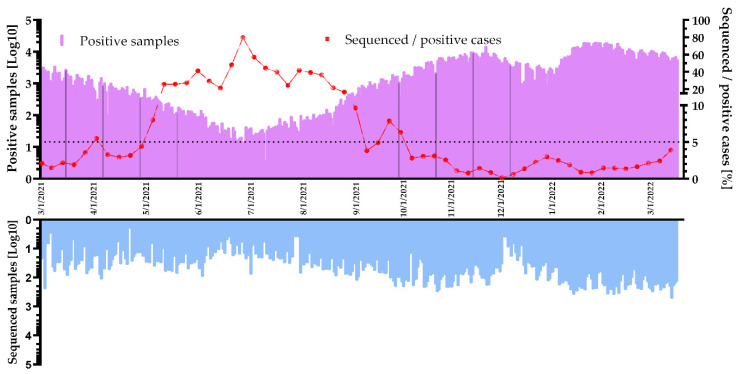
Number of positive and sequenced SARS-CoV-2 cases with proportion of coverage over time in SR. Daily numbers of positive tests (purple histogram), weekly coverage (red line) and sequenced samples (blue histogram) were changing during time with a peak in late 2021 and at the beginning of 2022.

Slovakia’s SARS-CoV-2 genomic surveillance strategy focused on collecting samples prepared by the state and private laboratories, hospitals, and from citizens returning from countries abroad where novel VOCs were announced. Genomic laboratories sequenced number of samples (from ca 40 to 1650 per week) according to epidemiological situation in that time. In June, July and August 2021 number of positive cases dropped, which also mirrored in sequencing volume. During this period, sequencing volume dropped on average to 100 samples per week. At the beginning of the year 2022, a novel VOC Omicron (B.1.1.529) and its derived subvariants started to circulate in our population with an unprecedented increase of new infections in the whole country. This situation led to an increase in sequencing numbers to over 1500 positive cases per week (Figure 2) necessitating a more integrated approach to data handling and analysis.

To estimate an actual prevalence of virus variants with sufficient precision, the volume of sequenced samples needs to achieve adequate levels, ideally 5% of all positive cases from the same period [14]. Therefore, we calculated the proportion of weekly sequencing volumes compared to weekly positive cases. In June, July and August 2021, the positive cases for SARS-CoV-2 infections dropped and the proportion of sequenced cases was over 40% and higher. At the beginning (March, April and May of 2021) and at the end of the analyzed period (October to December 2021 and January to March 2022), VOC Alpha (B.1.1.7), Delta (B.1.617.2) and Omicron (B.1.1.529), respectively, were dominant. Therefore, high numbers of positive cases caused a lower percentage of sequencing coverage, 2.35% on average (Figure 2).

### 3.4. Virus Variant and Lineage Prevalence

**Figure 3:**
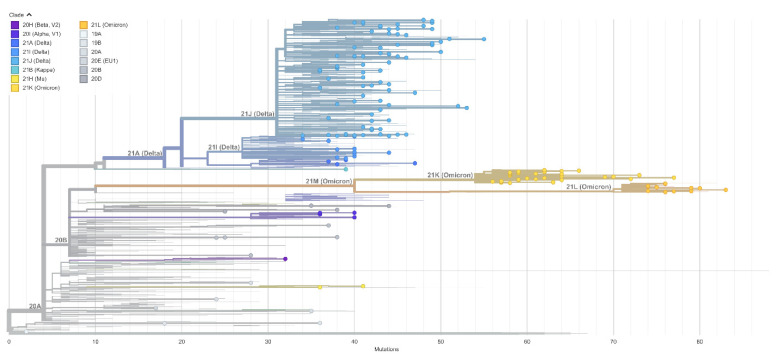
Evolutionary relationships of SARS-CoV-2 samples detected in SR. Altogether 165 different Pango lineages were detected in the evaluated dataset. Individual points in figure represents one lineage detected and deposited in GISAID database on the backbone of global evolutionary tree of SARS-CoV-2 virus.

**Figure 4:**
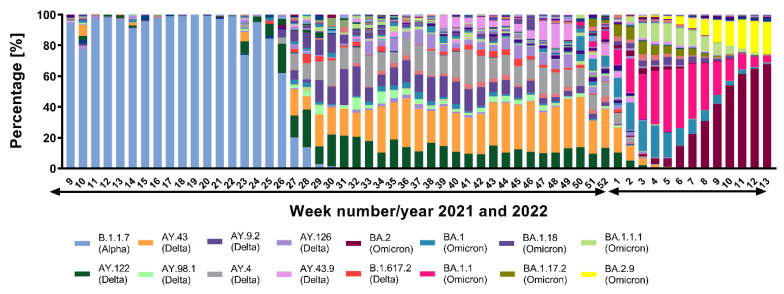
Development of SARS-CoV-2 lineages in SR. Weekly proportions of virus lineages started with dominant VOC Alpha (B.1.1.7) and later in week 26/27 (July/2021) with rapid onset of Delta (B.1.617.2) splitting into many lineages. New VOC Omicron (BA.1.1.529) began to outcompete Delta (B.1.617.2) in week 51/52 (December/2021) and quickly reaching prevalence and dominating until March/2022.

Each SARS-CoV-2 consensus sequence was analyzed with the GISAID Pangolin pipeline to generate the lineage when deposited in the GISAID repository. In our dataset we identified 165 unique lineages of the SARS-CoV-2 virus. These lineages are depicted as single points on the global phylogeny tree, representing all main variants since the pandemic started (Figure 3). The 16 most prevalent lineages detected with more than 1% proportion to all analyzed samples together with the rest of lineages are in Supplementary Table S2. The most prevalent lineage detected during the analyzed period was BA.2 (*n* = 4268 samples), present in 15.24% of SARS-CoV-2 positive cases followed by BA.1.1 (*n* = 3390 samples, 12.11%), B.1.1.7 (*n* = 3143 samples, 11.22%) and AY.43 (*n* = 3127 samples, 11.17%). We identified 4 out of 5 WHO variants of concern (VOC), Alpha (B.1.1.7), Beta (B1.351), Delta (B.1.617.2) and Omicron (BA.1.1.529). From the list of variants of interest (VOI), we were able to detect two variants, Kappa (B.1.617.1) and Mu (B.1.621). The prevalent VOC Alpha (B.1.1.7) was circulating in the Slovak population until the end of June 2021, followed by an increase of lineages AY.43, AY.4, AY.122 and AY.9.2 of the Delta (B.1.617.2) variant. Delta lineages were circulating in the Slovak population until the last weeks of 2021. The rapid start of the Omicron (BA.1.1.529) variant was detected at the beginning of 2022. Variant Omicron and its lineages continued to be prevalent until March, with dominant lineage BA.2 and BA.2.9 (Figure 4).

### 3.5. Sequence Quality of NGS Data

**Figure 5:**
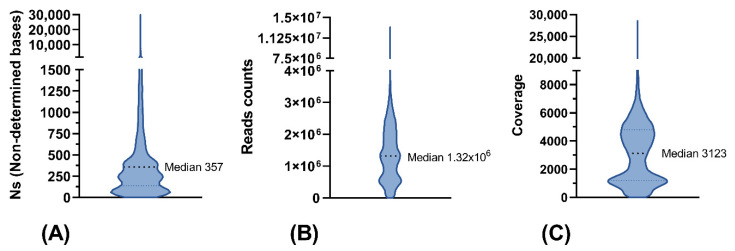
Summary of three analyzed NGS metrics. (**A**) Profile of undetermined bases with median value 357 (**B**) Profile of read counts with median value 1.32 × 10^6^ (**C**) Coverage of known part of the genome with median value 3123.

All samples were sequenced on second-generation sequencing platforms and all of them were made by Illumina. More than half of the sequence data, exactly 51.48% (*n* = 14,417) were generated with NextSeq 500 sequencer. The rest of the Illumina platforms were used as follows: MiniSeq 10.41% (*n* = 2915), MiSeq 11% (*n* = 3082) and NextSeq 550 27.11% (*n* = 7591). Three metrics were analyzed to evaluate and summarize quality of NGS data deposited in the public GISAID database. Since data were collected from four different sequencing platforms and initial quality and/or quantity of RNA was diverse, we decided to summarize these NGS metrics: Ns (non-determined bases), read count and mean coverage (Figure 5).

### 3.6. Genome Sequencing Centers Participating in the SARS-CoV-2 Genomic Surveillance Program

**Figure 6:**
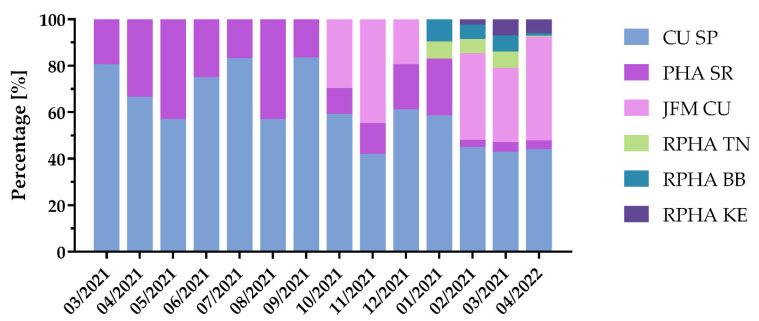
SARS-CoV-2 genomic centers. Total six genomic centers performed sequencing of analyzed with Illumina platforms. First half of analyzed period was caried out in SP CU and PHA in Bratislava. Second half of period was distributed to all six centers.

The complexity of the genome sequencing process and its specific requirements regarding next-generation sequencing platforms and extensive dataset processing were divided into several genomic centers. At the beginning, from March up to September, only two genomic centers, CU SP and PHA SR, were capable of Illumina based sequencing and analyzing of SARS-CoV-2 positive samples along with the Oxford Nanopore sequencing based laboratory at the Slovak Academy of Science. Later, additional genomic centers were set up at the end of the year 2021 and the beginning of 2022 (Figure 6). Overall, genomic center CU SP in Bratislava participated in sequencing of 52.73% of all samples (*n* = 33,024) sent within the analyzed period. Other genomic centers participated as follows: 26.04% by JFM CU in Martin, 12.1% by PHA in Bratislava, regional PHA in Trenčín, Banská Bystrica and Košice contributed by 3.22%, 3.49% and 2.41% of sequenced samples, respectively.

## 4. Discussion

This analysis of COVID-19 pandemic is focused mainly on genomic surveillance of SARS-CoV-2 virus in the SR carried out in participating Illumina technology-based laboratories under coordination by the Public Health Authority of the Slovak Republic and in the specific time interval. We used publicly available sequences generated in the SR with included metadata to summarize and describe the development of the first global pandemic in the 21st century in Slovak population (SR population 5.45 × 10^6^) [33].

Since the start of the COVID-19 pandemic, most countries have faced several significant waves of SARS-CoV-2 positive cases. Over time there was a gradual accumulation of differences such as the start of the outbreak, duration of the wave and type of variant responsible for it [34]. The first global wave occurred in the first months of 2020 after a new virus was described in Wuhan city, China, at the end of December 2019 [35]. The first cases in the European region were reported in France on 24 January 2020 [36] and the first positive case in SR was reported on 6 March 2020 [37]. The number of infected persons during the first global wave in the SR was under control thanks to strict government measures (for example, closed schools, declared a state of emergency). From March until October a total of 11,617 infections were confirmed by real-time PCR [37]. However, the situation deteriorated significantly from October 2020 with the new lineage B.1.1.7 detected in November 2020. B.1.1.7 probably originated in September 2020 in the South East region of England [38] and has caused a worldwide increase in cases, including Slovakia. Therefore, it was necessary to support the early detection of circulating variants and global efforts to monitor and evaluate the development of SARS-CoV-2 also in the SR.

Although the first isolates of the SARS-CoV-2 virus in SR were sequenced in March 2020 [39], the national sequencing program was not launched until March 2021. In addition to the PHA SR, another six sequencing laboratories participated in the national SARS-CoV-2 genomic surveillance, five implementing Illumina based sequencing—CU SP, JFM CU, RPHA BB, RPHA TN and RPHA KE and one implementing Oxford nanopore sequencing—Biomedical Center of Slovak Academy of Sciences.

Here we report the genomic data in GISAID database which were created by our team from data of all Illumina based sequencing laboratories between March 2021 to March 2022. In thirteen months, Illumina based SARS-CoV-2 sequencing laboratories contributed to public databases with 28,005 SARS-CoV-2 high-quality sequences and corresponding metadata, which is more than 86% of all genomes in GISAID from the Slovak Republic during the period. Before March 2021, in general, most countries did not reach high or medium percentages (0.1% to 1%) of sequenced cases each week of the pandemic [17]. In the study by Chan et al. [40], analyzing global data (data from 118 countries) of SARS-CoV-2 genome surveillance until 31 October 2021, SR was ranked among 45 countries with a high level of routine genomic surveillance.

We analyzed the period that encompasses three significant waves of SARS-CoV-2 VOCs. The first part of the data (1st March 2021 and later) represents positive samples from the Alpha variant (B.1.1.7) wave of COVID-19, ending in the middle of July 2021. This part of the wave was characterized by the lower number of daily positive SARS-CoV-2 cases compared to subsequent waves (total 81,098 SARS-CoV-2 positive tests; the highest daily cases: 1027/1 × 10^6^ people). However, the impact on total confirmed deaths was higher (*n* = 5136; highest daily new confirmed deaths: 18.8/1 × 10^6^ people) than total deaths during the Delta or Omicron waves. This situation was also underlined by the high number of hospitalized and intensive care unit (ICU) patients (highest daily occupation of hospitals or ICU with COVID-19 patients: 715 or 112/1 × 10^6^ people, respectively) [41].

The second wave of COVID-19 in SR was caused by the Delta (B.1.617.2) variant and its AY lineages. The first cases of the second wave were detected and sequenced in July 2021. These variants and their subvariants caused more SARS-CoV-2 positive cases in the population (*n* = 498,683) and fewer total deaths (*n* = 4932) when compared with previous analyzed period. The highest recorded statistics for daily positive cases were 2083/1 × 10^6^ people, 17.8/1 × 10^6^ people for daily confirmed deaths, 615/1 × 10^6^ people for patients occupying hospitals and 115/1 × 10^6^ people for patients occupying ICU [41].

The third wave of COVID-19 started with the first Omicron (B.1.1.529) cases in January 2022 following subsequent variants BA.1 and BA.2. Impact of the Omicron variant (until the end of March 2022) was different when compared with previous waves of COVID-19. During the two and half months, we observed over 800,000 cases of positive SARS-CoV-2 samples, with the highest daily positive cases 4148/1 × 10^6^ people. Such a high increase of infections in the population led to 1848 confirmed deaths, with the highest daily number 7.16/1 × 10^6^ people. Also, the burden of hospitals was lower, with the highest hospital and ICU patient occupancy at 534/1 × 10^6^ and 50/1 × 10^6^ people, respectively [41].

When we compare COVID-19 waves by using the ratio of confirmed SARS-CoV-2 positive patients to confirmed deaths, we noticed during the Omicron wave 443.6 positive cases per one death patient. However, confirmed death cases were much more frequent during the Delta and the Alpha waves, and thus 101.1 or 15.7 positive cases per one death patient, respectively. These differences can be explained by several factors which are constantly in flux. For example, changes in the transmissibility of different variants and the degree of their virulency. Evolution evasion to immunity gained by infection or vaccination, level of human countermeasures and their compliance through pandemic or percentage of fully vaccinated people [42,43].

It is obvious that proportion of sequenced samples varies through the time and it is influenced by the number of new COVID-19 cases. In months with the highest number of daily positive cases, the surveillance program allowed determination of variants of approximately 2% of samples. Despite original plans to sequence approximately 500 samples per week, this required number increased during Delta wave and even more during the Omicron wave. The rise of analyzed samples leads to bottlenecks in processing of samples, as well as in handling of metadata for uploads to repositories. This led to development of an integrated central information system, which allowed scheduling of Illumina sequencing laboratories, more robust and automated data and metadata transfer, unified analysis and automated batch database uploading. This system with abbreviated name NarCoS is operating since late February 2022. The system helped to increase the robustness, speed and accuracy to accommodate the need for the rapid increase of the number of sequenced samples.

## Data Availability

In this study we used the metadata openly available in GISAID database at (https://www.gisaid.org/) (accessed on 23 May 2022) [doi:10.17616/R3Q59F].

## Supplementary Materials

The following supporting information can be downloaded at: https://www.mdpi.com/article/10.3390/v14112432/s1, Table S1: Districts of SR with number of total sequenced positive SARS-CoV-2 samples.; Table S2: List of 165 lineages detected from 1 March 2021 up to 31 March 2022.
