# Supplementary material for: Systematic Genomic Surveillance of SARS-CoV-2 Virus on Illumina Sequencing Platforms in the Slovak Republic—One Year Experience
Source: Viruses. 2022 Nov 2;14(11):2432. doi: 10.3390/v14112432 (PMC9697771; doi:10.3390/v14112432)
Supplement: Supplementary file 1 [file viruses-14-02432-s001.zip › viruses-1984135-supplementary.pdf]

Supplementary Table S1. Districts of SR with number of total sequenced positive SARS-CoV-2 samples.

District	No. of samples	District	No. of samples	District	No. of samples	District	No. of samples
**Bratislava**	3894	**Myjava**	437	**Pezinok**	198	**Bánovce nad Bebravou**	82
**Poprad**	1808	**Brezno**	390	**Lučenec**	180	**Partizánske**	79
**Trenčín**	1726	**Martin**	387	**Považská Bystrica**	179	**Banská Štiavnica**	77
**Prešov**	1621	**Sabinov**	377	**Snina**	162	**Šaľa**	76
**Ilava**	1300	**Michalovce**	368	**Piešťany**	151	**Žarnovica**	74
**Košice**	1067	**Ružomberok**	356	**Žiar nad Hronom**	147	**Levoča**	68
**Trnava**	913	**Prievidza**	341	**Zvolen**	139	**Rožňava**	64
**Nitra**	705	**Tvrdošín**	340	**Sobrance**	133	**Bytča**	62
**Malacky**	641	**Námestovo**	327	**Topoľčany**	129	**Krupina**	60
**Banská Bystrica**	632	**Senica**	277	**Humenné**	128	**Zlaté Moravce**	58
**Komárno**	593	**Čadca**	255	**Bardejov**	127	**Rimavská Sobota**	57
**Žilina**	591	**Púchov**	255	**Kysucké Nové Mesto**	119	**Dolný Kubín**	52
**Trebišov**	580	**Kežmarok**	244	**Stropkov**	109	**Gelnica**	48
**Nové Zámky**	518	**Dunajská Streda**	240	**Nové Mesto nad Váhom**	107	**Turčianske Teplice**	48
**Galanta**	489	**Liptovský Mikuláš**	239	**Veľký Krtíš**	102	**Revúca**	38
**Skalica**	476	**Spišská Nová Ves**	239	**Svidník**	92	**Poltár**	29
**Košice - okolie**	474	**Levice**	215	**Vranov nad Topľou**	91	**Detva**	23
**Senec**	470	**Stará Ľubovňa**	210	**Hlohovec**	85	**Medzilaborce**	21

Supplementary Table S2. List of 165 lineages detected from March 1, 2021 up to March 31, 2022.

Lineage	No. of samples	Lineage	No. of samples	Lineage	No. of samples	Lineage	No. of samples	Lineage	No. of samples
**BA.2**	4268	**AY.5**	52	**AY.9**	15	**AY.3**	3	**B.1.617.1**	1
**BA.1.1**	3390	**BA.1.14**	52	**AY.113**	14	**AY.105**	3	**B.1.1**	1
**B.1.1.7**	3143	**AY.23**	50	**AY.41**	13	**AY.4.9**	3	**B.1.1.523**	1
**AY.43**	3127	**AY.84**	44	**BA.2.25**	13	**AY.5.4**	3	**B.1.621.1**	1
**AY.4**	1587	**AY.60**	44	**B.1.258**	12	**AY.33.2**	3	**Q.7**	1
**AY.122**	1500	**AY.70**	42	**AY.117**	11	**BA.1.1.16**	3	**B.1.619**	1
**BA.1**	1376	**BA.2.25.1**	42	**AY.120**	10	**BA.2.23**	3	**AY.69**	1
**BA.2.9**	1330	**AY.125**	39	**AY.98**	10	**B.1**	2	**AY.110**	1
**AY.9.2**	1007	**BA.1.1.4**	39	**AY.53**	9	**A**	2	**AY.54**	1
**BA.1.1.1**	994	**BA.1.15.1**	38	**AY.23.2**	9	**C.36**	2	**A.29**	1
**AY.126**	672	**BA.1.1.11**	35	**BA.1.13**	9	**B.1.621**	2	**AY.93**	1
**AY.43.9**	596	**AY.129**	34	**AY.33**	8	**AY.1**	2	**AY.37**	1
**BA.1.17.2**	502	**AY.94**	31	**AY.25.1**	8	**AY.46.4**	2	**AY.5.5**	1
**BA.1.18**	367	**AY.127**	30	**B.1.160**	7	**AZ.2**	2	**AY.4.14**	1
**AY.98.1**	290	**BA.1.8**	27	**B.3**	6	**AY.46.5**	2	**AY.16**	1
**B.1.617.2**	287	**BA.1.21**	27	**AY.44**	6	**AY.106**	2	**AY.128**	1
**AY.46.6**	271	**BA.2.5**	27	**AY.4.5**	6	**AY.100**	2	**AY.134**	1
**AY.42**	206	**AY.6**	26	**AY.121.1**	6	**AY.32**	2	**AY.46.2**	1
**BA.1.17**	206	**AY.4.7**	25	**BA.1.12**	6	**AY.34.1**	2	**AY.4.16**	1
**BA.1.15**	173	**BA.1.1.14**	25	**AY.103**	5	**AY.88**	2	**AY.39.1.1**	1
**AY.68**	159	**AY.20**	23	**AY.43.8**	5	**AY.36.1**	2	**AY.83**	1
**AY.46**	142	**AY.7.1**	22	**BA.1.21.1**	5	**AY.4.17**	2	**AY.51**	1
**AY.112**	140	**AY.20.1**	22	**BA.2.12**	5	**AY.4.2.2**	2	**AY.45**	1
**AY.4.4**	112	**AY.36**	22	**B.1.1.1**	4	**AY.43.4**	2	**AY.118**	1
**AY.39**	112	**BA.1.1.18**	20	**C.36.3**	4	**BA.1.10**	2	**AY.4.2.4**	1
**BA.2.3**	110	**B.1.351**	19	**AY.78**	4	**BA.1.1.13**	2	**AY.127.1**	1
**AY.121**	103	**AY.34**	19	**AY.75**	4	**BA.1.1.17**	2	**BA.1.1.2**	1
**BA.1.20**	102	**BA.2.1**	19	**AY.25**	4	**BA.1.16**	2	**BA.1.1.7**	1
**BA.1.19**	92	**AY.124**	18	**AY.87**	4	**BA.2.19**	2	**BA.1.9**	1
**AY.26**	81	**AY.4.2**	18	**AY.102**	4	**B.1.177.32**	1	**BA.1.1.12**	1
**AY.4.13**	76	**BA.1.1.15**	17	**Q.1**	3	**B.1.1.192**	1	**BA.1.1.10**	1
**BA.2.10**	72	**AY.4.6**	16	**B.1.1.170**	3	**AY.71**	1	**BA.1.5**	1
**AY.4.2.3**	66	**BA.1.6**	16	**AY.7.2**	3	**C.38**	1	**BA.2.32**	1
